# Fatty Acid Supplementation Reverses the Small Colony Variant Phenotype in Triclosan-Adapted *Staphylococcus aureus*: Genetic, Proteomic and Phenotypic Analyses

**DOI:** 10.1038/s41598-018-21925-6

**Published:** 2018-03-01

**Authors:** Abdulrahman S. Bazaid, Sarah Forbes, Gavin J. Humphreys, Ruth G. Ledder, Ronan O’Cualain, Andrew J. McBain

**Affiliations:** 1 Division of Pharmacy and Optometry, School of Health Sciences, Faculty of Biology, Medicine and Health, Stopford Building, The University of Manchester, Manchester, UK; 2 College of Applied Medical Sciences, University of Hail, Hail, Saudi Arabia; 3 Biomolecular Sciences Research Centre, Sheffield Hallam University, Sheffield, UK; 4 Biological Mass Spectrometry Core Facility, Faculty of Biology, Medicine and Health, The University of Manchester, Manchester, UK

## Abstract

*Staphylococcus aureus* can develop a small colony variant (SCV) phenotype in response to sub-lethal exposure to the biocide triclosan. In the current study, whole genome sequencing was performed and changes in virulence were investigated in five *Staphylococcus aureus* strains following repeated exposure to triclosan. Following exposure, 4/5 formed SCV and exhibited point mutations in the triclosan target gene *fabI* with 2/4 SCVs showing mutations in both *fabI* and *fabD*. The SCV phenotype was in all cases immediately reversed by nutritional supplementation with fatty acids or by repeated growth in the absence of triclosan, although *fabI* mutations persisted in 3/4 reverted SCVs. Virulence, determined using keratinocyte invasion and *Galleria mellonella* pathogenicity assays was significantly (p < 0.05) attenuated in 3/4 SCVs and in the non-SCV triclosan-adapted bacterium. Proteomic analysis revealed elevated FabI in 2/3 SCV and down-regulation in a protein associated with virulence in 1/3 SCV. In summary, attenuated keratinocyte invasion and larval virulence in triclosan-induced SCVs was associated with decreases in growth rate and virulence factor expression. Mutation occurred in *fabI*, which encodes the main triclosan target in all SCVs and the phenotype was reversed by fatty acid supplementation, demonstrating an association between fatty acid metabolism and triclosan-induced SCV.

## introduction

*Staphylococcus aureus* causes a wide range of community and hospital acquired infections^1^. Coupled with the colonisation of almost a third of the general population^2^, the ability to cause recurrent infections, and antibiotic resistance, *S. aureus* treatment is increasingly challenging. In some reports, recurrent *S. aureus* infections have been associated with the emergence of small colony variants (SCVs), a slow-growing subpopulation of bacteria^3,4^. Although there are conflicting reports about the antibiotic susceptibility and comparative virulence of *S. aureus* SCVs, reduced antibiotic susceptibility in SCVs associated with prolonged antibiotic therapy such as aminoglycosides has been reported^5^. In addition to reduced growth rates, the SCV phenotype may be modulated during infection and the expression of virulence factors regulated accordingly, thus promoting persistence and survival within host cells^6–9^.

According to previous reports, *S. aureus* can form SCVs following exposure to sub-lethal concentrations of the phenolic biocide, triclosan^10,11^, which is commonly used for disinfection and hygiene purposes. Triclosan has broad-spectrum antibacterial activity, with the major mode of action involving the disruption of fatty acid biosynthesis through the inhibition of the enoyl-acyl protein reductase enzyme FabI at bacteriostatic concentrations^12^. According to some reports, triclosan also directly targets the bacterial cell membrane^13,14^ although disruption in fatty acid synthesis may also indirectly affect cell membrane integrity.

The formation of gentamicin-induced *S. aureus* SCVs has been attributed to a mutation in the electron transport chain genes, *menD* and *hemB*^7^ resulting in auxotrophy for these growth factors. In contrast, triclosan-induced SCVs may not be deficient in any of these growth factors and as such their emergence could be due to impaired energy production^10^.

The chacteristics of triclosan-induced SCV have been previously investigated for *S. aureus* ATCC 6835^11^ where the SCVs showed reduced pathogenicity in an invertebrate model and exhibited decreased hemolysis, coagulase and DNase production^11^. This triclosan-induced SCV also exhibited defects in cell division overexpression of the triclosan target enzyme (FabI)^15^. However, whilst the emergence of *S. aureus* SCVs during antibiotic exposure has been previously investigated, comparatively little is known about the mechanisms resulting in the formation of SCVs in response to triclosan, or the implications of adaptation. The aim of the current study therefore was to explore genomic alterations and reversibility in triclosan-adapted *S. aureus* SCVs. Additionally, to expand the previous observations^11,15^, relative pathogenicity and cell invasion were assessed in a panel of SCVs and a non-SCV forming strain. Protein expression was also investigated to gain further insights into the phenotypic alterations associated with triclosan adaptation.

## Results

### Development of the SCV phenotype

After triclosan exposure (P10), 4/5 strains (ATCC 43300, NCTC 13277, SAR17 and Newman) formed the SCV phenotype as evidenced by the formation of pinpoint colonies. The colony morphology of strain SAR2831 remained unchanged. In all cases, the SCV phenotype was unstable, reverting following five or more passages in the absence of triclosan.

### Mutations in *S. aureus* SCVs

Single non-synonymous mutations in the *fabI* gene were observed in P10 and PX10 SCVs for Newman, NCTC13277 and SAR17 strains (Table 1), where cytosine (C) changed to thymine (T) leading to an amino acid change (A95V). This mutation was sustained when triclosan was removed (PX10) although compensatory mutations were not detected. ATCC 43300 exhibited a different *fabI* single nucleotide polymorphism (G113C). A mutation was also identified in the *fabD* gene of both ATCC 43300 (V111D) and the Newman strain (Q228K) following exposure to triclosan. The latter mutation was also observed in the Newman strain after recovery in the absence of triclosan (PX10). Teicoplanin resistance associated membrane gene (*tcaA*) exhibited a mutation (G373V) in the triclosan exposed non-SCV strain SAR2831which was also observed in PX10.

**Table 1:** Summary mutations and resulting amino acid changes in *S. aureus* strains after triclosan exposure.

Gene	Protein encoded	*S. aureus* strains									
		ATCC43300*		Newman*		NCTC13277*		SAR17*		SAR2831	
		P10	PX10	P10	PX10	P10	PX10	P10	PX10	P10	PX10
*fabI*	Enoyl-ACP reductase	G113C	—	A95V	A95V	A95V	A95V	A95V	A95V	—	—
*fabD*	Malonyl CoA-acyl carrier protein transacylase	V111D	—	Q228K	Q228K	—	—	—	—	—	—
*tcaA*	Teicoplanin resistance associated membrane	—	—	—	—	—	—	—	—	G373V	G373V
*NWMN_0826*	NADH-dependent flavin oxidoreductase	—	—	H107N	H107N	—	—	—	—	—	—

P0, bacteria before exposure to triclosan; P10, following ten passages with triclosan exposure; PX10, following further ten passages without triclosan exposure. A: alanine, N: asparagine, D: aspartic acid, C: cysteine, Q: glutamine, G: glycine, H: histidine, K: lysine. *SCV.

### Triclosan induced SCVs revert with fatty acid supplementation

**Figure 1:**
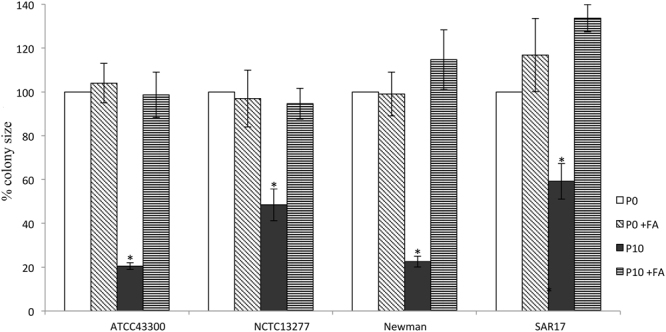
Colony size of P0 and SCVs (P10) with and without the addition of fatty acids. All P10 SCVs showed reduced colony size compared to P0. When the media supplemented with fatty acids, all SCV colony size reverted to pre-exposure size (P0). *Significant change (p < 0.05) compared to P0 and FA stands for media supplemented with fatty acids (Tween 80). Results are means and standard error from representative colonies (n = 3).

The Newman *S. aureus* strain and the SCV derived from ATCC43300 showed the largest reduction in colony size when comparing P10 to the original parent strains (21% and 24% respectively; p < 0.001) while SCV derived from SAR17 and NCTC13277 colonies were almost 50% (p < 0.02) the size of the wild-type strain. When SCVs were supplemented with fatty acids, they underwent full phenotypic reversion (p < 0.05) in the SCV phenotype (Fig. 1).

### Reduced triclosan susceptibility in *S. aureus* SCV

Table 2 illustrates the susceptibility of *S. aureus* to triclosan following triclosan exposure. Strains exhibiting the SCV phenotype (derived from ATCC 43300, NCTC 13277, SAR17 and the Newman strain) exhibited a significant decrease (p < 0.05) in sensitivity to triclosan at MIC and MBC. This contrasts with the non-SCV strain, SAR2831, which exhibited no significant change in either MIC or MBC to triclosan. This alteration in susceptibility was sustained even when triclosan was removed (PX10) in terms of both MIC and MBC.

**Table 2:** Minimum inhibitory concentrations and minimum bactericidal concentrations for triclosan before (P0), after exposure to triclosan (P10) and in the absence of the biocide (PX10).

Passage number	*S. aureus* strains									
	ATCC43300*		NCTC13277 *		Newman*		SAR17*		SAR2831	
	MIC	MBC	MIC	MBC	MIC	MBC	MIC	MBC	MIC	MBC
P0	2 (1)	12 (3)	5 (2)	12 (3)	1	13	1	25	6	25
P10	**12 (3)**	**38 (14)**	**17 (6)**	**38 (14)**	**6 (2)**	**42 (13)**	**10 (3)**	33 (13)	13	50
PX10	**12 (3)**	**33 (13)**	**12 (3)**	**42 (13)**	**13**	**46 (10)**	**9 (3)**	33 (13)	13	50

See footnote to Table 1. MIC and MBC in five *S. aureus* isolates (µg/ml); numbers in bracket are standard deviation. Asterisks indicate organisms that formed SCV following triclosan exposure. Bold text indicates a statically significant difference in susceptibility (P < 0.05) compared to P0. Results are means and standard deviation from two separate experiments with three technical replicates.

### Reduced relative pathogenicity in triclosan exposed isolates

**Figure 2:**
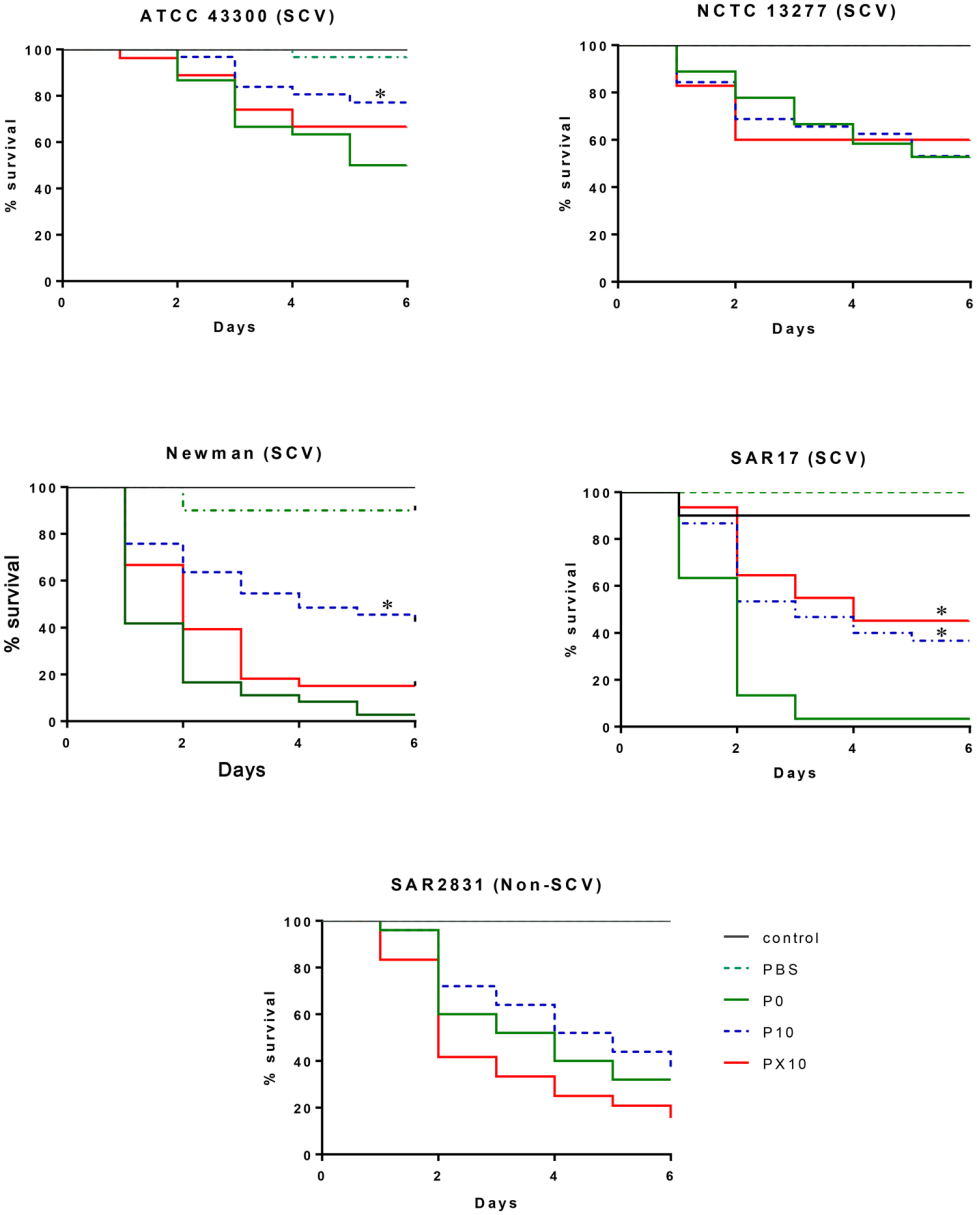
Survival curve of four SCVs (NCTC 13277, ATCC 43300, SAR17 and Newman) and SA2831 (non-SCV). All SCVs P10 strains exhibited reduced larval lethality compared to P0 with exception of NCTC 13277. ATCC 43300, Newman and SAR17 P10 exhibited a significant reduction in relative pathogenicity, and PX10 pathogenicity reverted to P0 level with exception of SAR17, which remain attenuated even when triclosan was removed (PX10). *Significant change (p < 0.05) compared to P0. Results are means from three separate experiments.

Results obtained from the relative pathogenicity assessment are shown in Fig. 2. Overall, 3/5 triclosan adapted *S. aureus* strains (ATCC 43300, Newman and SAR17) exhibited a reduced *G. mellonella* larvae virulence in comparison to their unexposed counterparts. Pathogenicity for SCV ATCC 43300 (p < 0.001), SAR17 (p < 0.0001) and Newman (p < 0.001) were significantly reduced when compared to the parent strains, which reverted to pre-exposure levels for Newman and ATCC43300 but not for SAR 17 following repeated growth in the absence of triclosan. NCTC 13277 (SCV) and SAR2831 (non-SCV) mutants did not exhibit any significant difference in pathogenicity when compared to their respective parent strain.

**Figure 3:**
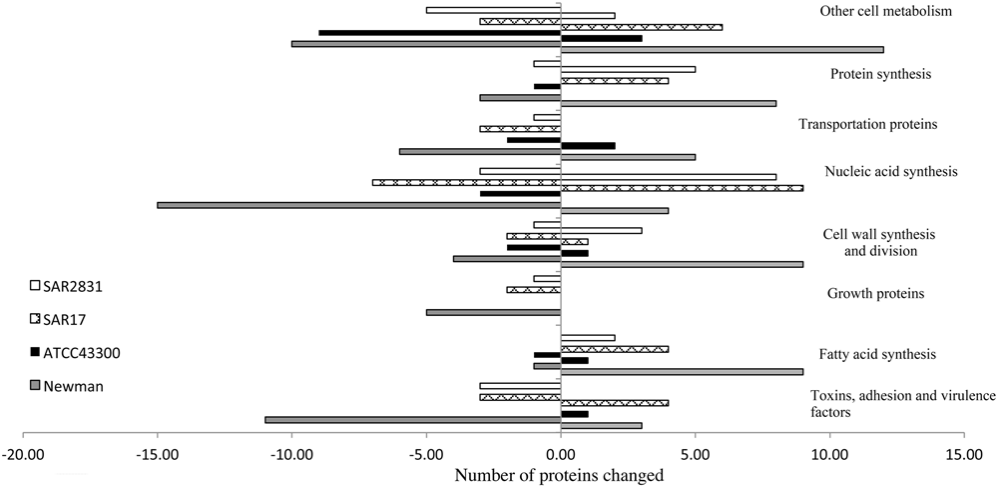
Proteins altered in 3 SCVs (Newman, ATCC 43300 and SAR17) and non-SCV (SAR2831) compared to P0 categorised based on the function or pathway. Newman exhibited the highest number in protein expression alteration while ATCC 43300 and SAR 17 showed changes in 26 and 48 proteins, respectively.

### Alterations in the *S. aureus* proteome following triclosan exposure

Proteins exhibiting a 2-fold or more significant change in expression were investigated for changes in the proteome. Following triclosan exposure, 50 proteins were significantly (p < 0.05) up-regulated and approximately 55 proteins (p < 0.05) were down-regulated in the Newman strain (Fig. 3). While in SAR17, 28 proteins showed increased expression and 20 showed downregulated expression. ATCC 43300 exhibited the lowest number of protein changes with 8 proteins becoming overexpressed and 18 under-expressed. In the non-SCV forming strain (SAR2831), 20 proteins were significantly up-regulated and approximately 15 proteins were down-regulated (Fig. 3). Protein expression data were categorised into eight groups based on protein function or pathway (Fig. 3). Although the majority of altered proteins in the PX10 isolates reverted to pre-exposure levels in Newman and ATCC 43300, SAR17 PX10 proteins failed to revert to P0 levels.

In terms of virulence associated proteins, in the Newman strain, proteins which play a role in *S. aureus* virulence, including response regulator SaeR, Immunodominant Antigen B, Gamma-Hemolysin Component A, Immunoglobulin-binding Protein sbi and Leukocidin/Hemolysin Toxin Family S, were down- regulated while response regulator protein GraR and drug resistance transporter EmrB/QacA were up-regulated. In SAR17, Immunoglobulin-Binding Protein sbi, Immunoglobulin G and Immunodominant Antigen B were upregulated; however, /Serine-Rich Adhesin for platelets SraP and Response Regulator Protein GraR were over-expressed. None of these proteins showed a significant change in ATCC 43300 except for EmrB/QacA that exhibited an up-regulation. In the non-SCVs (SAR2831) Gamma Haemolysin and Immunoglobulin G were downregulated.

In Newman and SAR17 (SCVs) as well as the non SCV bacterium SAR2831, fatty acid biosynthesis proteins were upregulated including the triclosan target enzyme enoyl-[acyl-carrier-protein] reductase FabI^12^ which was increased more than 4-fold, 13-fold and 6- fold, respectively. Several further fatty acids synthesis associated enzymes such as oxoacyl-[acyl-carrier-protein] synthase 2 were also up-regulated in both Newman and ATCC43300 strains (3.3 and 2.1-fold, respectively). The SAR17 and Newman SCV exhibited a greater than 2-fold increase in expression of oxoacyl-[acyl-carrier-protein] synthase 3 enzyme (FabH). Malonyl CoA-acyl transacylase, which is involved in fatty acid metabolism, was down regulated in both ATCC43300 and Newman after triclosan exposure (5 and 2.4-fold, respectively).

In the Newman strain, expression of stress-associated proteins such as threonine dehydratase II and pyruvate formate-lyase-activating enzyme were reduced. Alanine dehydrogenase was down- regulated in Newman, SAR17 and SAR2831 SCVs. Several peptidoglycan biosynthesis enzymes such as transglycosylase SceD and peptidoglycan hydrolase, as well as teichoic acid biosynthesis enzymes and cell membrane proteins were upregulated in the Newman SCV. Bifunctional autolysin was over-expressed in all strains except for ATCC43300. Transmembrane and cell division proteins were however, less expressed in all SCVs. Similarly, DNA synthesis and replication proteins exhibited a reduction in the SCVs as well as the non-SCV triclosan adapted strain. However, DNA binding and urease accessory proteins showed up-regulation following triclosan exposure in SCVs Newman and SAR17 and SAR2831. Transport proteins showed a variation in expression with the majority being downregulated while those associated with amino acids synthesis were overexpressed in all strains. SAR17, ATCC 43300 and SAR2831, showed an increase expression of 50 S ribosomal protein while carbamoyl-phosphate synthase was overexpressed in SAR 17. Several other cell metabolism proteins also exhibited different expression when exposed to triclosan (Supplement 1).

### Reduced keratinocyte invasion in triclosan exposed isolates

**Figure 4:**
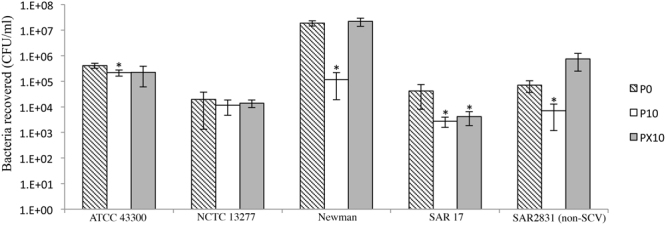
Keratinocytes invasion assay of P0, P10 and PX10 in four SCVs (NCTC 13277, ATCC 43300, SAR17 and Newman) and SA2831 (Non-SCV). ATCC 43300, SAR17, Newman and SAR2831 P10 exhibited a significant reduction in recovered bacteria and number of intracellular reverted to unexposed level when triclosan was removed. *Significant change (p < 0.05) compared to P0. Results are means and standard error from three separate experiments with two technical replicates.

SCVs, strain Newman (p < 0.001), ATCC 43300 (p < 0.01) and SAR17 as well as non-SCV SAR2831 (p < 0.05) showed a significant reduction in the number of recoverable bacteria after keratinocyte infection when compared to their unexposed counterparts (Fig. 4). In the recovered strains (PX10), the number of recoverable Newman and SAR 2831 reverted to unexposed levels however, SAR17 PX10 failed to revert to levels comparable to pre-exposure baselines (p < 0.05) (Fig. 4).

## Discussion

SCVs are a slow growing subpopulation of bacteria that can form in response to an environmental stresses including exposure to certain antibiotics and the biocide triclosan^16^. The induction of SCV by exposure to triclosan has been previously observed^10^ and we have reported attenuated virulence^11^ and reduced competitive fitness^15^ in SCV induced by this compound. The main purpose of the current investigation was to study the genomic changes underlying the formation and reversion of SCV following exposure to triclosan. In pursuing this aim we have expanded the panel of test bacteria used in previous studies to include clinical isolates and assessed the correlation between an invertebrate virulence assay and a keratinocyte invasion assay to assess the potential implications of the triclosan induced SCV. We have used high-resolution proteomics (LC-MS) to further investigate proteomic changes in SCV and established the SCV phenotype can be reversed by supplementation with fatty acids.

Five strains of *S. aureus* including MRSA were repeatedly exposed to sub-lethal concentrations of triclosan using a previously validated gradient plate method^11^. Following ten passages in the presence of triclosan, *S. aureus* strains demonstrated reduced triclosan susceptibility (Table 2) with 4/5 developing the SCV phenotype, suggesting that reduced triclosan susceptibility did not depend on the formation of SCVs. Reductions in triclosan sensitivity have been previously observed in other *S. aureus* SCVs^15^. At the genomic level a nonsynonymous *fabI* (A95V) mutation was identified in all the SCVs (Table 1) except for ATCC 43300 that instead exhibited (G113C) mutation in the *fabI* gene. This mutation was sustained when triclosan was removed (PX10) although compensatory mutations were not detected suggesting that with the SCV phenotype is not directly caused by *fabI* mutation or that phenotypic adaptation overcome the physiological effects of the mutation following repeated growth in the absence of the antimicrobial.

A (A95V) mutation in the FabI-triclosan binding site^17^ has been previously reported in triclosan adapted *S. aureus*^18^ and may contribute to the decrease in triclosan susceptibility (Table 2). According to some previous reports, in lipid-rich environments, Gram positive bacteria can overcome fatty acid inhibition by utilizing exogenous fatty acids^19^. We therefore investigated whether the SCV phenotype could be reversed by lipid supplementation. Interestingly, all SCV colonies increased in diameter to pre-exposure size immediately during growth with fatty acid supplementation (Fig. 1), suggesting that triclosan-induced SCVs are deficient in fatty acid synthesis. Since triclosan is lipophilic it is notable that reversal of the SCV phenotype occurred in adapted bacteria growing in the absence of triclosan and thus, partitioning of the antimicrobial into the supplemental lipids is unlikely to be responsible.

Despite their association with persistent infections, reductions in virulence have been previously reported in SCVs generated following antibiotic exposure^6–9,20^ or through sublethal exposure to the biocide triclosan in *S. aureus* ATCC 6835^11^. In the current study, relative pathogenicity was further investigated in a collection of triclosan adapted SCV, as well as non-SCV forming strain. In agreement with a previous investigation^11^, *S. aureus* SCVs exhibited a reduction in relative pathogenicity, evidenced by a reduced *G. mellonella* virulence when compared to parent strains (Fig. 2). *G. mellonella* is a useful model to investigate *S. aureus* pathogenesis and virulence^21^ because it has an innate immune system^22^. Moreover, SCVs have been previously associated with recurrent and persistent infections and have been shown to exhibit an increased ability to adhere to and invade host cells in addition to a reduced antibiotic susceptibility^8^. To gain further insight into the pathogenic potential of the adapted bacteria the ability of triclosan induced *S. aureus* to invade primary human keratinocytes was assessed, showing a reduction in recoverable bacteria in 3/4 SCVs as well as in the non-SCV forming strain (Fig. 4). Reduced intracellular persistence may be attributed to down regulation in virulence proteins (immunoglobulin-binding protein sbi) as well as a reduced growth rate evidenced by down expression in nucleic acids synthesis and cell division proteins (FtsL and FtsZ) (Supplement 1). Moreover, reduction in cell division protein (Supplement 1) may account for formation of the SCV phenotype and suggest that the bacteria are defective in cell division which correlates with our previously reported TEM analysis^15^.

SCV persistence has been associated with overexpression of fibronectin-binding protein FnBP^8^. In the current study, low FnBP expression in Newman SCV may explain the reduction in intercellular persistence because (FnBP) is crucial for bacterial host cell invasion^23^. FnBP also participates in adhesion formation (as reviewed by Josse *et al*.)^24^, therefore, a reduction in FnBP expression in triclosan adapted SCVs may partly explain impaired biofilm formation observed previously^11^. Newman SCV also exhibited low expression in both response regulator SaeR and histidine protein kinase SaeS proteins (Supplement 1), which are regulated by the *Sae* gene^25^. *Sae* controls the expression of many virulence genes such as *fnbA* and *hIg*, encoding fibronectin-binding and gamma-hemolysin proteins, respectively^26^. These proteins were down regulated in our Newman strain (Supplement 1). Down regulation of *sae* has been reported in *hemB* deficient SCVs^27^, thus attenuated virulence in our Newman SCV may be due to a down regulation in the *sae* gene. Moreover, 2 /4 SCV exhibited down-regulation of immune response proteins, immunodominant antigen B (Supplement 1), which could explain the reduced relative pathogenicity in triclosan-induced SCVs.

Proteomic data revealed an upregulation in the triclosan target enzyme FabI in two SCVs as well as the non-SCV forming strain (Supplement 1). This may be attributed to non-synonymous mutation in *fabI* in response to triclosan exposure (Table 1). Formation of such a mutation is proposed to be a compensatory mechanism whereby *S. aureus* attempts to compensate for fatty acid depletion^28^. Moreover, cell membrane proteins exhibited up-regulation, such as, EmrB/QacA a membrane fusion efflux pump protein, suggesting a possible link between efflux pump expression and decreased triclosan susceptibility in *S. aureus*.

Although SAR2831 did not exhibit the SCV phenotype following ten passages of triclosan exposure, there was a significant reduction in the number of recoverable bacteria in the keratinocyte invasion assay (Fig. 4) as well as a down-regulation in fibrinogen-binding protein FIB_STAAE (Supplement 1). Proteins associated with energy production (nucleoside diphosphate kinase and riboflavin biosynthesis protein) (Supplement 1) were down regulated in the non-SCV suggesting that reduced invasion observed in this bacterium might partially be attributed to reduced cell metabolism. This suggests that triclosan exposure effects *S. aureus* pathogenicity regardless of the formation of the SCV phenotype potentially through the down-regulation of the proteins. SAR2831 (non-SCV) showed a mutation in *tcaA* (teicoplanin resistance associated membrane protein) despite no difference being detected in MICs to teicoplanin before and after exposure to triclosan (data not shown).

## Conclusion

In this investigation, all triclosan-induced SCVs exhibited mutation in *fabI*, which encodes the Enoyl-[acyl-carrier-protein] reductase, the major target for triclosan. Triclosan-exposed *S. aureus* showed reduced pathogenicity and keratinocyte invasion regardless of the SCV phenotype, which may be attributed to a reduction in the expression of cell adhesion-associated proteins following triclosan adaptation. The potential involvement of deficiencies in fatty acid biosynthesis in triclosan SCV was evidenced by the reversal of the phenotype through fatty acid supplementation although *fabI* mutations persisted following reversion.

## Materials and Methods

### Bacterial strains and growth conditions

Five different *S. aureus* strains were used in this study. Two clinical wild type isolates obtained from the Molecular Diagnostics and Personalised Therapeutics Unit at the University of Hail (Hail, Saudi Arabia). Strain ATCC 43300 (MRSA) was acquired from the American Type Culture Collection while strains Newman and NCTC 13277 (MRSA) were supplied by Public Health England (Salisbury, UK). Bacteria were maintained on Tryptone Soya Agar (TSA) and Tryptone Soya Broth (TSB) which were purchased from Oxoid (Basingstoke, UK) and were sterilised at 121 °C and 15 psi for 15 minutes before use.

### Whole genome sequencing and data analysis

DNA extraction and whole genome sequencing (WGS) of P0, P10 and PX10 was provided by MicrobesNG using Illumina HiSeq platform. Sequences (length 2 × 250 bp paired-end reads) were analysed through several pipelines; starting by using Kraken to identify the closest reference genome, which confirmed that all sequences are *S. aureus*. The data were then *de novo* assembled using the SPAdes (http://bioinf.spbau.ru/spades) followed by variant calling against the closest reference genome (MRSA252 -UID57839, Newman-UID58839, ECT R2 -UID159389 and T0131-UID159861 for ATCC 43300, NCTC 13277, Newman, SAR17 and SAR2831, respectively). Nonsynonymous mutations were identified by comparing P10 and PX10 genomes to the untreated parent isolate (P0).

### Exposure to sub-lethal concentrations of triclosan and fatty acid supplementation

Reproducible concentration gradients of triclosan (100 µg/ml to 10 mg/ml) were spread on TSA using a Wasp II spiral plater (Don Whitley, Shipley, United Kingdom)^11,15^ and allowed to dry for 30 min at room temperature. Following this, overnight suspensions of *S. aureus* were radially inoculated onto plates in triplicate and incubated for 4 days (37 °C). After 4 days, a heavy inoculum was taken from the edge of the sub-lethal zone and inoculated onto a fresh plate containing the same (or higher) concentration of triclosan. This was passaging process was repeated a further ten times to produce P10. Following this, a further ten passages were performed in the absence of triclosan to create PX10. All stocks were cryopreserved at −80 °C for further testing. For the fatty acid supplementation experiment, MHA was supplemented with Tween 80 (0.1%) and plates were incubated at 37 °C for 24 h. After sample incubation, the plates were photographed, and images were imported into the ImageJ software. Three random colonies diameter were measured, and data presented as a percentage to pre-exposure strain (P0). Parent strains were also incubated with and without fatty acids to ensure that the increase in the colony size is not due to a simple nutritional response.

### Minimum Inhibitory Concentrations and Minimum Bactericidal Concentrations

MICs were determined using the broth microdilution assay as described previously^29^. Briefly, overnight bacterial cultures were diluted in TSB to an OD600 of 0.8 and then further diluted 1: 100 in TSB. Adjusted cultures were then transferred to a 96 well microtitre plate containing doubling dilutions of triclosan. The microdilution plates were incubated at 37 °C for 24 h. The MIC was defined as the lowest concentration of the antimicrobial that inhibits the bacterial growth. Bacterial growth was determined by visual inspection for turbidity in the well. Aliquots (5 μL) were transferred from wells showing no growth and placed into sterile TSA plates and incubated at 37 °C (24 h) to measure MBCs. MBC was defined as the lowest concentration of the antimicrobial required to show no growth after 4 days of incubation.

### *Galleria mellonella* pathogenesis assay

The pathogenesis assay was adapted from^11^. Final stage *G. mellonella* larvae were purchased from Live Foods Direct (Sheffield, United Kingdom) and stored in dark for a maximum of 7 days. 10 larvae (15–25 mm in length) were selected for each condition (P0, P10 and PX10). Overnight *S. aureus* cultures were grown in TSB and washed twice in sterile Phosphate Buffer Solution (PBS) then diluted to OD_600_ 0.3. Hamilton syringe were used to injected hemocele larvae through left proleg with the washed bacterial suspension (5 µl) (0.8–2.6 × 10^6^ CFU/Larvae which confirmed colony count in TSA). The syringe was washed by ethanol (100%) twice before injecting another group. Each group (10 larvae) were placed in sterile petri dish and incubated at 37 °C. A control group (not injected) and a PBS injected group were also incubated as a negative control. Survival of each group were recorded daily for 6 days and larvae considered dead when appear black or no response seen when touch. The experiment was performed three independent times and if two or more of the control larvae died, the experiment was repeated again. Results were presented in survival curve and log-rank test were calculated using Prism v.7 (www.graphpad.com/).

### Keratinocyte invasion assay

Invasion assays were performed as described by^30^. Normal primary human epidermal keratinocytes (NHEK) were purchased from Promocell (Germany) and were grown in 24 well-plate containing keratinocyte growth medium-2 (1 ml) (Promocell, Germany) until 90% confluence (3–5 × 10^5^ cells/well). Overnight *S. aureus* suspension was diluted in keratinocyte growth medium-2 to OD_600_ 0.4 (1–2.5 × 10^8^ CFU/ml). Bacteria were added at a keratinocyte: bacteria ratio of 1:100 and incubated for 2 h at 37 °C. Keratinocytes were washed twice with sterile PBS (1 ml) followed by addition of gentamicin (100 µl/ ml) and lysostaphin (10 µl/ml) to kill any unattached or loosely adhered bacteria. To lyse keratinocytes, distilled water (100 µl) and trypsin (100 µl) were added and serial dilutions of the lysed suspensions were plated on TSA, before incubation overnight at 37 °C and determining of remaining viable bacteria. Assay was performed in triplicate for each isolate (P0, P10 and PX10) and student t-test was used to determine significance between recoverable levels of bacteria.

### Protein extraction and preparation for mass spectrometry

All chemicals used were from Sigma Aldrich (Dorset, UK) unless otherwise stated. SCV that exhibited attenuated relative pathogenicity and significant reduction in recovered bacteria, were included for further protein analysis. Six replicates of P0, P10 and PX10 were grown in TSB overnight (37 °C, 100 Samples were prepared for prepared for mass spectrometry using a modified Filter-aided sample preparation (FASP) method^31^ with the following modifications: The cultures were pelleted by centrifugation at 4000 rpm for 10 minutes, the supernatant was decanted and the pellet was washed twice in PBS containing 1% protease inhibitors. The washed pellet was resuspended in 1 mL of PBS with lysostaphin (10% v/v) and kept on ice for 15 minutes. The washed cells were transferred to Zirconia/Silica bead-containing tubes (Stratech Scientific, UK) and lysed in a FastPrep 120 (Thermo Scientific, UK) at 40 rpm for 20 seconds, with 20 seconds cooling, for a total of 3 cycles. Sodium Dodecyl Sulphate (SDS) (1%) and dithiothreitol (DTT) were added to a final concentration of 1% (w/v) and 50 mM respectively. The samples were incubated at 90 °C for 5 minutes. The lysate was centrifuged at 10,000 *g* for 5 minutes and the supernatant was transferred to clean tube and the protein concentration was measured using a Direct Detect Spectrometer (Merck Millipore).

For FASP, clarified lysate (25 µg) was added to 100 µL of wash buffer (8 M urea, 0.1 Tris-HCl (pH 8.5) with 15 mM DTT) and washed twice in a 30 kDalton filter (Merck Millipore). To alkylate the sample, 50 µL of buffer (8 M urea, 0.1 M Tris-HCl (pH 8.5) with 0.05 M iodoacetamide) was added to the filter and the sample was incubated in darkness at room temperature for 30 minutes. The filters were washed twice with washing buffer 1 followed by a further two washes of washing buffer 2 (6 M urea, 0.1 Tris-HCl (pH 8.5) with 25% deionised water) before dilution in 50 mM Tris-HCl (pH 8.5) to bring the urea concentration to 1 M. Samples were digested by the addition of trypsin at a protein: enzyme ratio of 1:100 and incubated overnight at 37 °C. After digestion, peptides were collected by centrifugation at 4000 *g* at 20 °C for 15 minutes and the filtration units were washed once with washing buffer 2 and subsequently with two washes of 40 mM ammonium bicarbonate. Peptide samples were transferred to 96 well plate with 0.2 µM PVDF membrane (Corning) and desalted with OLIGO R3 reversed-phase media (Applied Biosystems). Desalted peptides were dried down and resuspended in 5% acetonitrile with 0.1% formic acid and analysed by LC-MS/MS using an UltiMate® 3000 Rapid Separation LC (RSLC, Dionex Corporation, Sunnyvale, CA) coupled to an Orbitrap Elite (Thermo Fisher Scientific, Waltham, MA) mass spectrometer. Peptide mixtures were separated using a gradient from 92% A (0.1% FA in water) and 8% B (0.1% FA in acetonitrile) to 33% B, in 44 min at 300 nL min-1, using a 250 mm × 75 μm i.d. 1.7 mM BEH C18, analytical column (Waters). Peptides were selected for fragmentation automatically by data dependant analysis (DDA).

The acquired MS data was analysed using Progenesis LC-MS (v4.1, Nonlinear Dynamics). The retention times in each sample were aligned using one LC-MS run as a reference, then the “Automatic Alignment” algorithim was used to create maximal overlay of the two-dimensional feature maps. Features with charges ≥+ 5 were masked and excluded from further analyses, as were features with less than 3 isotope peaks. The resulting peak lists were searched against the UniProt database with *Staphylococcus aureus* (Strain Newman) sequences included (UniProt, download date: 2016/02/26) using Mascot v2.5.1, (Matrix Science). Search parameters included a precursor tolerance of 5 ppm and a fragment tolerance of 0.5 Da. Enzyme specificity was set to trypsin and one missed cleavage was allowed. Carbamidomethyl modification of cysteine was set as a fixed modification while methionine oxidation was set to variable. The Mascot results were imported into Progenesis LC-MS for annotation of peptide peaks. Proteins exhibiting 2 or more-fold significant changes (at a p < 0.05) were included in the study.

### Data availability

All data generated or analysed during this study are included in this published article (and its Supplementary file).

## Electronic supplementary material

Supplementary information.
