# Supplementary material for: Fatty Acid Supplementation Reverses the Small Colony Variant Phenotype in Triclosan-Adapted Staphylococcus aureus: Genetic, Proteomic and Phenotypic Analyses
Source: Sci Rep. 2018 Mar 1;8:3876. doi: 10.1038/s41598-018-21925-6 (PMC5832852; doi:10.1038/s41598-018-21925-6)
Supplement: Supplementary file 1 — Supplementary information. [file 41598_2018_21925_MOESM1_ESM.docx]

**Fatty Acid Supplementation Reverses the Small Colony Variant Phenotype in Triclosan-Adapted *Staphylococcus aureus*: Genetic, Proteomic and Phenotypic Analyses**

**Abdulrahman S Bazaid^a,b^, Sarah Forbes^c^, Gavin J Humphreys^a^**

**Ruth G Ledder^a^, Ronan O'Cualain^d^ and Andrew J. McBain^a*^**

^a^Division of Pharmacy and Optometry, School of Health Sciences,

Faculty of Biology, Medicine and Health, Stopford Building,

The University of Manchester, Manchester, UK.

^b^College of Applied Medical Science, University of Hail, Hail, Saudi Arabia.

^c^Biomolecular Sciences Research Centre, Sheffield Hallam University, Sheffield, UK.

^d^Biological Mass Spectrometry Core Facility, Faculty of Biology, Medicine and Health, The University of Manchester, Manchester, UK

*For correspondence: Andrew J McBain, Division of Pharmacy and Optometry, Faculty of Biology, Medicine and Health, The University of Manchester, Oxford Road, Manchester M13 9PT, UK. Tel: 44 161 275 2360; Email: andrew.mcbain@manchester.ac.uk

| **Supplement 1**: Summary of protein expressions in P10 and PX10 compared to P0 (in fold change) and their function. Minus numbers indicate down-regulated protein while positive numbers mean increase in expression. | | | | | | | | | | |
| --- | --- | --- | --- | --- | --- | --- | --- | --- | --- | --- |
| **Accession** | Name | Newman | | ATCC 43300 | | SAR 17 | | SAR2831 | | Function/ pathway |
|  |  | P10 | PX10 | P10 | PX10 | P10 | PX10 | P10 | PX10 |  |
| **Virulance factors** | | | | | | | | | | |
| A0A0H3KAA5_STAAE | Gamma-hemolysin component A | -42.5 | -27.9 | - | - | - | - | - | - | Toxin that forms pores in the cell membrane |
| A0A0D6HCK9_STAAU | Gamma-hemolysin component B | - | - | - | - | - | - | -2.4 | -1.1 | Toxin that forms pores in the cell membrane |
| A0A0H3KDS7_STAAE | Sensor protein VraS | -12.8 | -13.7 | - | - | - | - | - | - | transmembrane signalling systems |
| SAES_STAAE | Histidine protein kinase SaeS | -11 | -14.4 | - | - | - | - | - | - | sensing protein |
| SBI_STAAE | Immunoglobulin-binding protein sbi | -16 | -13 | - | - | -4.8 | -3.9 | - | - | protecting bacteria against host immune response |
| A0A0U1MFS0_STAAU | Immunoglobulin G-binding protein A | - | - | - | - | -4.4 | -4.7 | -3.5 | -3.8 | protecting bacteria against host immune response |
| FIB_STAAE | Fibrinogen-binding protein | -14 | -53 | - | - | - | - | -4.5 | -2.3 | Binds to host fibrinogen |
| A0A0H3KFE3_STAAE | Lipase | -7 | -3.3 | - | - | - | - | - | - | Breakdown host lipid. |
| SCIN_STAAE | Staphylococcal complement inhibitor | -6.3 | -7.1 | - | - | - | - | - | - | inactivate human neutrophils opsonization |
| SAER_STAAE | Response regulator SaeR | -5.8 | -1.7 | - | - | 2 | 3 | - | - | control adhesion and invasion of host cell |
| A0A0H3KAT9_STAAE | Immunodominant antigen B | -2.7 | -2.2 | - | - | -2 | -2 | - | - | virulence factor that induce Immune response |
| A0A0H3KHT5_STAAE | Leukocidin/hemolysin toxin family S | -3.6 | -17 | - | - | - | - | - | - | staphylococcal hemolysin toxin |
| A0A0H3K9Y8_STAAE | Staphylococcal accessory regulator V | -2 | -1.6 | - | - | - | - | - | - | control adhesion to host cell |
| SDRE_STAAE | Serine-aspartate repeat-containing protein E | 3.4 | 1.5 | - | - | - | - | - | - | involved in the aggregation of host platelets |
| A0A0D6DQ91_STAAU | Serine-rich adhesin for platelets SraP | - | - | - | - | 6 | 9 | - | - | involved in the aggregation of host platelets |
| GRAR_STAAE | Response regulator protein GraR | 2 | 1.4 | - | - | 2.5 | 2.8 | - | - | resistance against cationic antimicrobial peptides |
| A0A0H3KA69_STAAE | Drug resistance transporter EmrB/QacA | 2.3 | 1.7 | 4 | 1.9 | - | - | - | - | activate efflux pump |
| A0A0D1HPY5_STAAU | Accessory gene regulator A | - | - | - | - | 18 | 13 | - | - | binding to the RNAIII-agr regulatory region |
| **Fatty acids synthesis** | | | | | | | | | | |
| A0A0H3K7V7_STAAE | Enoyl-[acyl-carrier-protein] reductase [NADPH] | 4.1 | 9 | - | - | 13 | 36 | 6.1 | 14 | fatty acid biosynthesis |
| A0A0H3KBU2_STAAE | 3-oxoacyl-[acyl-carrier-protein] synthase 2 | 3.3 | 2.5 | 2.1 | 1.7 | - | - | - | - | Catalyzes fatty acid synthesis |
| FABH_STAAE | 3-oxoacyl-[acyl-carrier-protein] synthase 3 | 3 | 2.3 | - | - | 2.3 | 3 | - | - | Catalyzes fatty acid synthesis |
| A0A069FYU7_STAAU | 3-hydroxyacyl-[acyl-carrier-protein] dehydratase FabZ | - | - | - | - | 2.9 | 5 | - | - | unsaturated fatty acids biosynthesis |
| A0A0H3KEC0_STAAE | Aldo/keto reductase family protein | 3 | **1.1** | - | - | - | - | 3.5 | 5.7 | Catalysis oxidation-reduction (redox) reaction |
| Y695_STAAE | Putative lipid kinase | 2.4 | 1.1 | - | - | - | - | - | - | catalyze lipids phosphorylation |
| A0A0H3KCE2_STAAE | 3-oxoacyl-[acyl-carrier protein] reductase | 2.2 | 1.7 | - | - | - | - | - | - | fatty acid biosynthesis |
| PLSX_STAAE | Phosphate acyltransferase | 2.9 | 2 | - | - | - | - | - | - | fatty acid biosynthesis |
| ACCD_STAAE | Acetyl-coenzyme A carboxylase carboxyl transferase | 2.1 | 1.3 | - | - | 5.4 | 4 | - | - | involved in Lipid metabolism |
| A0A0H3K8B9_STAAE | Glycerol-3-phosphate dehydrogenase | 2.6 | 1.9 | - | - | - | - | - | - | Lipid metablosim |
| A0A0H3K8D2_STAAE | Malonyl CoA-acyl carrier protein transacylase | -2.7 | -1.6 | -5.6 | -5 | - | - |  |  | malonyl metabolism |
| **Growth proteins** | | | | | | | | | | |
| A0A0H3KCR5_STAAE | Alanine dehydrogenase | -18 | -16 | - | - | -2.3 | -3.4 | -4.5 | -15 | catalyse the formation of pyruvate in anaerobic condition |
| A0A0H3K8M5_STAAE | Threonine dehydratase II | -16 | -16 | - | - | - | - | - | - | Catalysis of the reaction: L-threonine in anaerobic conditions |
| A0A0H3K744_STAAE | Regulatory protein Spx | -2.3 | -1.7 | - | - | - | - | - | - | control growth in stress |
| A0A0H3K7N9_STAAE | CsbD-like superfamily protein | -3.1 | -2.9 | - | - | -9 | -6 | - | - | stress protein |
| A0A0H3KDM3_STAAE | Pyruvate formate-lyase-activating enzyme | -2.4 | -2.2 | - | - | - | - | - | - | Activation of pyruvate formate-lyase under anaerobic conditions |
| **cell wall synthesis and division proteins** | | | | | | | | | | |
| SCED_STAAE | Probable transglycosylase SceD | 4 | -1.3 | 1.6 | 2.4 | - | - |  |  | cleave peptidoglycan and affects separation of bacterial cells |
| A0A0H3K6J4_STAAE | Peptidoglycan hydrolase | 3.7 | **-1** | - | - | - | - | - | - | hydrolysis bonds within peptidoglycan |
| Y1738_STAAE | UPF0754 membrane protein | 3.3 | 2.2 | - | - | - | - | - | - | Cell membrane synthesis |
| A0A0H3K7R3_STAAE | Transcription factor FapR | 2.3 | 1.9 | - | - | - | - | - | - | Involved in regulation of membrane lipid biosynthesis |
| SEPF_STAAE | Cell division protein SepF | 2.4 | 1.5 | - | - | - | - | - | - | cell division protein |
| A0A0H3K7X7_STAAE | Bifunctional autolysin | 2.3 | 1.5 | - | - | 2.5 | 4 | 2.2 | 3.4 | Cleaves the peptidoglycan to complete cell division cycle. |
| A0A0H3KBB7_STAAE | Teichoic acid biosynthesis protein D | 2.1 | 1.8 | - | - | - | - | - | - | Teichoic acid biosynthesis |
| A0A0H3KFB2_STAAE | D-alanine lipoteichoic acid | 2.2 | 1.6 | - | - | - | - | - | - | Teichoic acid biosynthesis |
| A0A0H3KIE3_STAAE | Cell envelope-related transcriptional attenuator | 2.1 | 1.1 | - | - | - | - | - | - | Part of putative membrane-bound proteins |
| A0A0H3K7N2_STAAE | Cell division protein FtsL | -3.7 | -1.8 | -2.1 | -2.3 | - | - |  |  | Essential cell division protein |
| A0A0D1FG35_STAAU | Cell division protein FtsZ | - | - | - | - | -2.2 | -2.7 | - | - | Essential cell division protein |
| A0A0D1I8M9_STAAU | Cell division protein FtsK | - | - | - | - | - | - | 2.6 | 3.6 | Essential cell division protein |
| A0A0H3KB41_STAAE | N-terminal deoxyribonuclease | -2 | -2 | - | - | - | - | - | - | Transmembrane protein |
| A0A0H3K692_STAAE | Sodium:dicarboxylate symporter family protein | -6.7 | -2.4 | - | - | - | - | - | - | transmembrane protein |
| A0A069G604_STAAU | GTP-binding protein EngB | - | - | - | - | -8 | -8 | - | - | cell division |
| A0A0H3KJ61_STAAE | N-acetylmuramoyl-L-alanine amidase | - | - | -2.5 | -2.7 | -2.3 | -2.4 |  |  | peptidoglycan synthesis |
| A0A0H2CDC1_STAAU | Cell wall surface anchor family protein | - | - | - | - | - | - | -5.4 | -13 | peptidoglycan synthesis |
| A0A0E1XHC1_STAAU | Adenylate cyclase | - | - | - | - | - | - | 2.3 | 8.2 | plasma membrane synthesis |
| **Nucleic acid synthesis and replication** | | | | | | | | | | |
| A0A033UUD6_STAAU | Inosine-5'-monophosphate dehydrogenase | - | - | - | - | -75 | -100 | - | - | plays an important role in the regulation of cell growth. |
| A0A033UT10_STAAU | DNA gyrase subunit A | - | - | - | - | 54 | 27 | - | - | supercoils double-stranded (ds) DNA in an ATP-dependent manner. |
| A0A0D6GXM9_STAAU | DNA RNA helicase | - | - | - | - | 12 | 8 | - | - | essential for DNA replication |
| A0A0B6XN16_STAAU | DNA-binding protein | - | - | - | - | -37 | -58 | -3.3 | -10 | DNA replication |
| A0A033UBD0_STAAU | Arginine--tRNA ligase | - | - | - | - | -7 | -9 | - | - | RNA synthesis |
| A0A0H3K643_STAAE | Purine nucleoside phosphorylase DeoD-type | -11.3 | -5.7 | -3.1 | -2.5 | - | - |  |  | purine nucleoside metabolic process |
| A0A0H3K7Y5_STAAE | Phosphoribosylamine--glycine ligase | -5.9 | -2.2 | - | - | -2.1 | -4 | - | - | Purine metabolism |
| PYRB_STAAE | Aspartate carbamoyltransferase | -3.7 | -1.7 | - | - | - | - | - | - | Pyrimidine metabolism |
| PURL_STAAE | Phosphoribosylformylglycinamidine synthase subunit PurL | -3.3 | -1 | - | - | - | - | - | - | purines biosynthetic pathway |
| PUR5_STAAE | Phosphoribosylformylglycinamidine cyclo-ligase | -3.2 | -1.1 | - | - | - | - | - | - | purines biosynthetic pathway |
| A0A0H3K5Z1_STAAE | 5'-nucleotidase, lipoprotein e(P4) | -3.2 | -3.2 | - | - | - | - | - | - | acid phosphatase activity |
| A0A0H3K8S5_STAAE | DNA primase | -3 | -3.1 | - | - | - | - | - | - | DNA replication |
| A0A0H3KC25_STAAE | Phosphoribosylglycinamide formyltransferase | -3 | -1 | - | - | -2.2 | -5 | - | - | Purine metabolism |
| A0A0H3K7C2_STAAE | Phosphoribosylformylglycinamidine synthase subunit PurQ | -2.6 | -1 | -2.3 | -2.9 | -2 | -4 |  |  | Purine metabolism |
| A0A0H3KCC6_STAAE | Orotate phosphoribosyltransferase | -2.3 | -1.8 | - | - | - | - | - | - | Pyrimidine metabolism |
| PYRF_STAAE | Orotidine 5'-phosphate decarboxylase | -2.1 | -1.7 | - | - | - | - | - | - | Pyrimidine metabolism |
| PUR7_STAAE | Phosphoribosylaminoimidazole-succinocarboxamide synthase | -2.1 | 1.4 | - | - | - | - | - | - | Purine metabolism |
| A0A0H3KFM7_STAAE | N5-carboxyaminoimidazole ribonucleotide synthase | -2.6 | 1.2 | - | - | 4 | 9 | - | - | Purine metabolism |
| TRMHL_STAAE | Putative TrmH family tRNA/rRNA methyltransferase | -2.4 | -2.1 | - | - | - | - | - | - | RNA synthesis |
| A0A0H3K7P6_STAAE | Dihydroorotase | -3.2 | -1.7 | - | - | - | - | - | - | Pyrimidine metabolism |
| A0A0H3K7M2_STAAE | Cold-shock protein CSD | - | - | -2.6 | -2.7 | - | - | -2.3 | 2.1 | DNA binding |
| UREG_STAAE | Urease accessory protein UreG | 3.4 | 1.3 | - | - | 6.8 | 13 | 4.1 | 4.4 | promote the incorporation of urease nickel metallocenter |
| A0A0H3K763_STAAE | UvrB/UvrC motif domain protein | 3 | 1.8 | - | - | - | - | - | - | DNA binding protein |
| UNG_STAAE | Uracil-DNA glycosylase | 2.1 | 1.9 | - | - | - | - | - | - | Excises uracil residues from DNA |
| EX7L_STAAE | Exodeoxyribonuclease 7 large subunit | 2.2 | 1.5 | - | - | 6 | 5 | - | - | DNA degradation |
| A0A0Z4SD81_STAAU | RNA methyltransferase TrmA family | - | - | - | - | 15 | 20 | - | - | RNA binding protein |
| A0A0H2IIF7_STAAU | Urease subunit beta | - | - | - | - | 9 | 15 | - | - | urea degradation |
| A0A077W0W4_STAAU | Urease subunit gamma | - | - | - | - | 19 | 37 | 7.2 | 11 | urea degradation |
| A0A0D1I3J5_STAAU | Urease accessory protein UreE | - | - | - | - | - | - | 7.7 | 13 | urea degradation |
| A0A0Z1INW2_STAAU | tRNA methyltransferase | - | - | - | - | - | - | 2.4 | 1.3 | biosynthesis of the modified nucleoside |
| A0A0H2HWA8_STAAU | DNA ligase | - | - | - | - | - | - | -4.8 | -2.4 | DNA synthesis |
| A0A0H2HUB6_STAAU | Putative competence-damage inducible protein | - | - | - | - | - | - | 2.4 | 3.6 | involve in horizontal transfer of genes |
| A0A0Z5B427_STAAU | RpiR family transcriptional regulator | - | - | - | - | - | - | 2.3 | 1.7 | control transccription |
| A0A069FS33_STAAU | Thioredoxin reductase | - | - | - | - | - | - | -2.3 | -2.1 | control transccription |
| A0A123C3S6_STAAU | N6-adenine-specific DNA methylase | - | - | - | - | - | - | 2.3 | 3.3 | protect DNA against degradation by restriction enzymes |
| A0A0H2JRS5_STAAU | Proline-tRNA ligase | - | - | - | - | - | - | 2.2 | 3.3 | RNA synthesis |
| **transport proteins** | | | | | | | | | | |
| A0A0H3KDK9_STAAE | Glutamine transport ATP-binding protein | 2.2 | 1.6 | - | - | - | - | - | - | transport system for glutamine |
| A0A0H3KBH8_STAAE | Di-/tripeptide ABC transporter | 2.1 | 1.2 | - | - | -6.5 | -4 | - | - | peptide transporter |
| A0A0H3KA74_STAAE | CorA-like Mg2+ transporter protein | 2 | 1.3 | - | - | - | - | - | - | magnesium transporter |
| A0A0H3K9X7_STAAE | Protein translocase subunit SecY | - | - | 2.3 | 2.1 | - | - | - | - | protein transport |
| A0A0H3KBC1_STAAE | Ferrichrome transport ATP-binding protein fhuA | - | - | 2.3 | 2 | - | - | - | - | ATP transport |
| A0A0H3KAK3_STAAE | ABC transporter, ATP-binding protein | -8.8 | -2.2 | -2.1 | -2.3 | -3.7 | -1.9 | - | - | ATP transport |
| A0A0H3K6N8_STAAE | ABC transporter, ATP-binding MsbA | -6.1 | -3.1 | - | - | - | - | - | - | ATP transport |
| A0A0H3KEU6_STAAE | ABC transporter ATP-binding protein | -3 | -1.3 | - | - | - | - | -4.2 | -1.4 | ATP transport |
| A0A0H3KII2_STAAE | L-lactate permease 2 | -3.2 | -1.3 | - | - | - | - | - | - | lactate transmembrane transporter |
| LDH2_STAAE | L-lactate dehydrogenase 2 | -3 | -2.4 | - | - | - | - | - | - | lactate transmembrane transporter |
| A0A0H3K9X9_STAAE | L-lactate permease | -3 | -1.8 | - | - | - | - | - | - | lactate transmembrane transporter |
| A0A0E1AJV5_STAAU | Copper ion binding protein | - | - | - | - | -7.6 | -5 | - | - | Copper transport |
| COPZ_STAAE | Copper chaperone CopZ | - | - | -3.1 | -3.1 | - | - | - | - | Copper transport |
| **protein synthesis** | | | | | | | | | | |
| A0A0D1HJY4_STAAU | Amino-acid citrate synthetase | - | - | - | - | 13 | 13 | - | - | Amino-acid biosynthesis. |
| A0A0H3KEK5_STAAE | Protein-arginine kinase | 3.8 | 2.3 | - | - | - | - | 2.5 | 2.7 | phosphorylation of arginine residues in proteins synthsis |
| A0A0H3KAT3_STAAE | Carbamate kinase | 3.7 | 16.1 | - | - | - | - | - | - | arginine synthesis |
| ARCA_STAAE | Arginine deiminase | 3.2 | -1.7 | -2.3 | -2 | - | - | 2.8 | 3.1 | arginine synthesis |
| A0A0H3KH72_STAAE | Mannosyl-glycoprotein endo-beta-N-acetylglucosamidase | 2.4 | 1 | - | - | - | - | - | - | Amino-acid biosynthesis. |
| A0A0H3KI69_STAAE | Alpha-acetolactate synthase | 2.3 | 1.2 | - | - | - | - | - | - | Amino-acid biosynthesis. |
| ASSY_STAAE | Argininosuccinate synthase | 2.1 | 1.1 | - | - | 4 | 5 | 2.6 | 2.6 | Amino-acid biosynthesis |
| METE_STAAE | 5-methyltetrahydropteroyltriglutamate methyltransferase | 2 | 1.8 | - | - | - | - | - |  | Amino-acid biosynthesis |
| ILVC_STAAE | Ketol-acid reductoisomerase | 2.6 | 1 | - | - | - | - | - | - | Amino-acid biosynthesis |
| RL28_STAAE | 50S ribosomal protein L28 | - | - | 2.2 | 2.7 | 3.4 | 4 | 7 | 11 | protein synthesis |
| A0A0H3K852_STAAE | Amino acid permease | -36 | -29 | - | - | - | - | - | - | Enables the transfer of amino acids from through membrane |
| CARB_STAAE | Carbamoyl-phosphate synthase large chain | -2.2 | -1.1 | - | - | 2.2 | 3 | -6.3 | -4 | Amino-acid biosynthesis. |
| A0A0H3K8B6_STAAE | Carbamoyl-phosphate synthase small chain | -2.3 | -1.7 | - | - | - | - | - | - | Amino-acid biosynthesis. |
| A0A0D1HVY3_STAAU | Nitroreductase | - | - | - | - | - | - | 2.3 | 2.4 | protein synthesis |
| **other cell metabolism** | | | | | | | | | | |
| A0A0H3KJ30_STAAE | Glycine betaine aldehyde dehydrogenase | 3.2 | 1.9 | 2.1 | 2.3 | - | - |  |  | Amine and polyamine biosynthesis |
| A0A0H3KFD1_STAAE | Chaperone protein ClpB | 2.6 | 1.1 | - | - | 4.3 | 1.5 | 6.5 | 3 | preventing proteins aggregation in response to heat |
| A0A0H3KIR5_STAAE | Alkyl hydroperoxide reductase AhpD | 2.7 | 1.3 | - | - | - | - | - | - | Antioxidant protein |
| GLPK_STAAE | Glycerol kinase | 2.5 | 1.5 | - | - | - | - | - | - | regulation of glycerol uptake and metabolism |
| A0A0H3KA40_STAAE | Abortive infection protein | 2.4 | 1.4 | - | - | - | - | - | - | resist bacteriophage infection |
| A0A0H3KDI8_STAAE | Indole-3-pyruvate decarboxylase | 2.6 | 1.4 | - | - | - | - | - | - | pyruvate metabolism |
| KCY_STAAE | Cytidylate kinase | 2.1 | 1.1 | - | - | - | - | - | - | energy production enzyme |
| A0A0H3KA34_STAAE | Inositol monophosphatase | 2.1 | 1.7 | - | - | - | - | - | - | signaling pathway |
| A0A0H3K8A3_STAAE | Riboflavin biosynthesis protein | 2 | 1.6 | - | - | 5 | 5.9 | -3.4 | -2.1 | energy production enzyme |
| A0A0H3KDQ2_STAAE | 2-C-methyl-D-erythritol 4-phosphate cytidylyltransferase | 2 | 1.1 | - | - | - | - | - | - | Isoprenoid biosynthesis |
| A0A0H3K8Q1_STAAE | Isocitrate dehydrogenase [NADP] | 2 | 1.1 | - | - | - | - | - | - | energy production enzyme |
| DNAJ_STAAE | Chaperone protein DnaJ | 2.1 | 1.6 | - | - | - | - | - | - | preventing proteins aggregation in response to heat |
| A0A0H3KB76_STAAE | Pyridine nucleotide-disulfide oxidoreductase | NS | NS | 2.2 | 2 | - | - |  |  | energy production |
| A0A0H3KFP0_STAAE | Cytochrome D ubiquinol oxidase | NS | NS | 2.2 | 3 | - | - |  |  | oxidation-reduction process |
| A0A0H3K8Q4_STAAE | Pyruvate kinase | -3.4 | -1.3 | - | - | - | - | - | - | synthesizes pyruvate in glycolysis pathway |
| A0A0H3K6Y2_STAAE | Alkyl hydroperoxide reductase subunit C | -2.3 | -2.8 | - | - | -2.8 | -2.2 | - | - | oxidative response protein |
| A0A0H3K760_STAAE | amino-hydroxy hydroxymethyldihydropteridine | -5.1 | -2.8 | - | - | - | - | - | - | energy production enzyme |
| A0A0H3K6G9_STAAE | Formate acetyltransferase | -2.7 | -2.1 | - | - | - | - | - | - | carbohydrate metabolic process |
| SCDA_STAAE | Iron-sulfur cluster repair protein ScdA | -3.2 | -2 | - | - | -2.7 | -1.7 | -5 | -5.3 | repair of iron-sulfur clusters |
| A0A0H3K988_STAAE | Ferritin | -4.8 | -3.8 | - | - | - | - | - | - | Iron-storage protein |
| A0A0H3K9P4_STAAE | Thiol-disulphide oxidoreductase, DCC family protein | -4.5 | -2.5 | - | - | - | - | - | - | oxidoreductase enzyme |
| A0A0H3KA53_STAAE | Esterase-like protein | -2.2 | -1.3 | - | - | - | - | - | - | hydrolysis enzyme |
| A0A0H3KDE4_STAAE | Aldehyde-alcohol dehydrogenase | -7.8 | -4.1 | - | - | - | - | - | - | carbon utilization |
| A0A0H3KB93_STAAE | Alcohol dehydrogenase | -3.9 | -3.5 | - | - | - | - | -2.1 | -2.9 | zinc metabolism |
| OTC_STAAE | Ornithine carbamoyltransferase | - | - | -5 | -2.3 | 5.4 | 6.6 | - | - | arginine biosynthesis |
| A0A0H3K7W9_STAAE | Glycosyl transferase | - | - | -3.7 | -4.1 | 3.1 | 2.9 | - | - | carbohydrate synthesis |
| A0A0H3K767_STAAE | Serine acetyltransferase | - | - | -2.8 | -2 | - | - | - | - | cysteine and sulfur metabolism |
| A0A0H3K814_STAAE | Amidophosphoribosyltransferase | - | - | -2.5 | -2.3 | - | - | - | - | formation of phosphoribosylamine |
| A0A0H3K8Q6_STAAE | Alpha-amylase | - | - | -2.3 | -2.7 | - | - | - | - | carbohydrate metabolism |
| A0A0H3K6G3_STAAE | Haloacid dehalogenase-like hydrolase | - | - | -2.2 | -2.1 | - | - | - | - | hydrolysis enzyme |
| A0A0H3K8E4_STAAE | Cytidine deaminase, homotetrameric | - | - | -2.1 | -1.9 | - | - | - | - | Zinc metabolism |
| A0A0H3KAA0_STAAE | Uroporphyrin-III C-methyl transferase | - | - | -2.1 | -1.8 | - | - | - | - | oxidation-reduction process |
| A0A0H3K9W5_STAAE | HAD-superfamily hydrolase subfamily IIB | - | - | -2.1 | -1.8 | - | - | - | - | hydrolase enzyme |
| A0A0D1GYY0_STAAU | 2-amino-4-hydroxy-6-hydroxymethyldihydropteridine diphosphokinase | - | - | - | - | -24 | -3 | - | - | energy production enzyme |
| A0A033V8D4_STAAU | Bifunctional protein FolD | - | - | - | - | 11 | 12 | - | - | carbon metabolism |
| A0A0E1XEQ3_STAAU | 2-succinylbenzoate--CoA ligase | - | - | - | - | 10 | 9 | - | - | synthesis of menaquinone |
| A0A0Z2XVS9_STAAU | Dehydrosqualene desaturase | - | - | - | - | 11 | 7 | - | - | staphyloxanthin synthesis |
| A0A033V5Q0_STAAU | Putative cysteine ligase BshC | - | - | - | - | - | - | 2.4 | -2.2 | maintenance of cellular redox balance |
| A0A0D1HQY5_STAAU | Nucleoside diphosphate kinase | - | - | - | - | - | - | -2.4 | -8.4 | control extracellular ATP |
| A0A0H2CU79_STAAU | Phosphoenolpyruvate-protein phosphotransferase | - | - | - | - | - | - | 2.2 | 3.8 | sugar phosphorylation |
